# Interleukin-6 to identify mildly injured patients in the trauma resuscitation room – a clinical feasibility study

**DOI:** 10.1007/s00068-026-03160-1

**Published:** 2026-03-24

**Authors:** Tim Niklas Bewersdorf, Marc Wimmer, Erik Popp, Jan Streblow, Lukas Baumann, Christine Leowardi, Stephan Stein, Gerhard Schmidmaier, Tobias Grossner

**Affiliations:** 1 Faculty of Medicine, Heidelberg University, 69120 Heidelberg, Germany; 2 Heidelberg Trauma Research Group, Clinic for Trauma and Reconstructive Surgery, Centre for Orthopaedics, Trauma Surgery and Spinal Cord Injury, Heidelberg University Hospital, 69120 Heidelberg, Germany; 3 Department of Anaesthesiology, Medical Faculty Heidelberg, Heidelberg University, 69120 Heidelberg, Germany; 4 Institute of Medical Biometry, Heidelberg University, 69120 Heidelberg, Germany; 5 Department of General, Visceral, and Transplantation Surgery, Heidelberg University Hospital, 69120 Heidelberg, Germany

**Keywords:** Interleukin-6, IL-6, Injury severity score, Trauma resuscitation room, Mildly injured trauma patients, Moderately injured trauma patients

## Abstract

**Purpose:**

Overtriage in the trauma resuscitation room (TRR) is often a consequence of admission based solely on trauma mechanism criteria. As interleukin-6 (IL-6) can assess injury severity in critically injured patients, the hypothesis of this study was that IL-6 can differentiate upon admission between mild (Injury Severity Score (ISS) 0–8) and moderate (ISS 9–15) trauma and that the parameter correlates with injury severity in lower injury severity groups (ISS < 16) during the first 24 h. Subsequently, we evaluated the ability to define IL-6 cut-off values with an adequate sensitivity, specificity, and negative predictive value to distinguish between moderate and mild injuries.

**Methods:**

Fifty patients admitted to the TRR solely based on trauma mechanism criteria and with an ISS < 16 were included in the study. IL-6 was measured at admission, 1, 6, 12 and 24 h later.

**Results:**

Thirty-one patients were mildly injured (ISS 0–8), while nineteen patients sustained moderate injuries (ISS 9–15). IL-6 correlated significantly with ISS at all time points. Moderately injured patients showed significantly higher IL-6 levels than mildly injured patients during the first 6 h. IL-6 levels at admission predicted moderate injuries for the IL-6 cut-off value of 2.9pg/ml with a sensitivity of 0.933, specificity of 0.370 and a negative predictive value of 0.970 resulting in undertriage rates of 6.6% and overtriage rates of 54.8%.

**Conclusion:**

The data of this feasibility study indicates that IL-6 has the potential to be employed in clinical workflows for early determination between patients with mild and moderate injuries, who are admitted to TRR based on trauma mechanism criteria to abort as early as possible TRR treatment and therefore free resources immediately while preserving patients’ safety.

**Supplementary Information:**

The online version contains supplementary material available at 10.1007/s00068-026-03160-1.

## Introduction

Trauma remains the leading cause of death in patients aged under 45 [1, 2]. To achieve the best possible outcome in potentially severely injured trauma patients, their treatment requires 24/7 availability of highly skilled interdisciplinary trauma teams, state-of-the-art trauma resuscitation rooms (TRR), operating theatres and intensive care units (ICUs) [3]. During the last decades the injury severity of patients treated in these centres has continuously decreased while at the same time there has been a significant increase in patients with only mild injuries who were falsely admitted to TRR resulting in an even higher redundant strain of the available resources [3–6].

Various guidelines, like the American College of Surgeons Committee on Trauma (ACS-COT) guideline or the German Trauma Society (DGU) S3 polytrauma guideline, give recommendations, primarily based on the clinical appearance of the patient, but secondarily also on the mechanism of injury, when to admit a patient to the TRR [1, 5–9]. According to the ACS-COT an undertriage rate of 5% and a overtriage rate of 50% seems acceptable regarding mindful management of available resources [8, 9]. Gianola et al. conducted a meta-analysis of several pre-hospital triage tools and revealed undertriage rates ranging between 3.6% and 66.8% and overtriage rates between 3.0% and 82.8%, but none fulfilled the ACS-COT recommendations [8]. Several studies have shown that especially trauma mechanism-based criteria are not a reliable predictor for higher injury severity, but cause significant overtriage of up to 76% if these criteria are applied [10–14]. Although various guidelines shrank trauma mechanism-based criteria for trauma team activation (TTA), overtriage is common in trauma care systems around the world and stresses the resources of trauma centres globally as these patients, admitted through the TRR commonly receive the full diagnostic algorithm including focused assessment with sonography for trauma (FAST), full body CT scan and often also monitoring at an ICU for 24 h even there is not always a clear medical indication for this. Furthermore, once a patient is admitted to a TRR downgrading- or abort protocols are often complex and require extended diagnostics to safely downgrade patients to the regular emergency department to ensure no severe injury has been missed. The ongoing discussion about TTA criteria for optimal triage rates and feasible downgrading- or abort protocols indicates the need for an additional method to identify mildly injured patients to support decision making if it would be justifiable to discontinue TRR treatment at an early timepoint to reduce unnecessary exhaust of resources [8]. Clinical assurance for an early abort of TRR treatment would already be beneficial as it would free resources immediately which would already be effective even if the overall number of TRR would not be directly reduced.

One of the most common scores to assess anatomical injury severity is the injury severity score (ISS) developed by Baker et al. [15]. Because mortality rates jump at distinct ISS, it is recommended to stratify patients into different ISS groups [16]. According to Rozenfeld et al. injury severity should be graded by ISS into four groups: 0–8, 9–15, 16–24, 25–75 [16]. From a clinical point of view differentiation between anatomically mildly (ISS 0–8) and moderately (ISS 9–15) injured trauma patients within the initial diagnostic phase in the TRR is essential, as moderately injured patients have a doubled mortality rate in comparison to mildly injured patients, and can suffer from more severe injuries (maximum abbreviated injury scale (MAIS): 3) like intestinal perforation, abdominal bleeding or open book fractures without haemorrhage, which need immediate diagnostics and therapy to avoid transformation into life-threatening injuries or complications, like haemorrhagic shock, organ failure or sepsis [1, 16–18]. However, it should be emphasized that the ISS is an anatomical injury scoring system that is usually calculated retrospectively and primarily used to stratify mortality risk [15, 16]. From a clinical standpoint, a retrospectively determined ISS of 0–8 does not in itself imply that the patient was overtriaged at the time of admission to the TRR. Clinical presentation on arrival, anatomical type and location of injuries, and relevant comorbidities associated with a high clinical risk may well justify TTA, TRR treatment, and subsequent ICU admission. Therefore, clinical injury severity assessment is multifactorial and based on several findings. Multiple attempts have been made in the past to rapidly determine trauma severity using various methods. The implementation of a quick biomarker test, that sensitively differentiates between different injury severity groups upon hospital admission would rule out moderate or high anatomical injury severity and could support precise identification of patients who have only mild or moderate injuries to endorse an early abort/discontinuation of TRR.

Interleukin-6 (IL-6) is a well-established biomarker, primarily involved in the acute phase response triggered by tissue damage [19]. As a systemic inflammatory response marker, IL-6 is a well-established early marker for bacterial infections and sepsis in critically ill patients [20]. Several studies in the past showed that IL-6 levels have a high predictive value for development of complications like sepsis, multi organ dysfunction syndrome (MODS) or multi organ failure (MOF) and mortality in at least moderately injured patients (ISS ≥ 9) [21–25]. Besides this, Gebhard et al. showed that IL-6 concentration strongly correlates with injury severity among patients who survived trauma as early as first blood sampling at the accident scene [26].

Although several studies revealed a strong correlation of IL-6 levels and injury severity, data is missing about early IL-6 level correlation with injury severity in only mildly (ISS 0–8) to moderately (ISS 9–15) injured patients, as other studies did not evaluate this patient cohort separately. Therefore, its efficacy for early differentiation between mild or moderate trauma remains unexplored. If IL-6 also correlates with injury severity in mildly and moderately injured trauma patients, who are frequently admitted to the TRR based on trauma mechanism criteria or preclinical provider’s decision, the implementation of such a biomarker could substantially improve resource management in trauma care. IL-6 could serve as a supportive early marker to identify mildly injured and thus potentially overtriaged patients who may not have a clear medical indication for full TRR treatment, although the final decision must always be based on the patient’s overall clinical condition and appearance. Therefore, any validated tool supporting clinical decision making regarding an early abortion of the TRR treatment would be highly valuable, as it would allow immediate reallocation of resources by transferring overtriaged patients to regular emergency department care.

Thus, we conducted a prospective, monocentric pilot study at a German level-I-trauma-centre with the hypothesis that the acute phase parameter IL-6 is able to differentiate upon admission between mild (ISS 0–8) and moderate (ISS 9–15) trauma and that the parameter correlates with injury severity in lower injury severity groups (ISS < 16) during the first 24 h. Furthermore, an assessment was performed to determine IL-6 cut-off values for a potential future clinical implementation to rule out moderate anatomical injuries. Here, the focus was on high sensitivity and a robust negative predictive value (NPV) to grant a significant impact upon under- and overtriage rates.

## Patients and methods

### Prevalence of trauma mechanism and moderately injured patients in the TRR

To calculate the prevalence of patients, who were admitted to the TRR solely based on trauma mechanism criteria, and the prevalence of moderately injured patients within this group, we analysed all TTAs between January 2018 and December 2021. All patients admitted to TRR based on trauma mechanism criteria without any injury related TTA, as displayed in Table 1, were included in this analysis. Injury severity was assessed after TRR treatment and diagnostics were completed. ISS was calculated as per standard of care in the TRR by a board-certified trauma surgeon. Based on ISS we differed between mildly (ISS: 0–8), moderately (ISS: 9–15), and severely injured trauma patients (ISS ≥ 16) [16].

**Table 1:** List of criteria for trauma team activation (TTA) sorted by injury- or trauma mechanism-related TTA. Displayed criteria were defined by German polytrauma standard care guidelines of German Trauma Society (DGU) in 2016

Injury-related trauma team activation (patients who met one of these criteria were excluded from the study)	Trauma mechanism-related trauma team activation (patients must show one of these criteria for inclusion)
Systolic blood pressure < 90 mmHg after trauma*	Fall from ≥ 3 m
Patients intubated or patients who have respiratory compromise*	Motor vehicle accident with: - Ejection from the vehicle - Delta velocity of > 30 km/h - Vehicle body intrusion of more than 50–75 cm - Collision of pedestrian or (motor)cyclist with car/truck - Death of a passenger of the same vehicle
Gunshot wound of torso/neck*	
GCS < 9 after trauma*	
Penetrating injury of torso/neck	
Fractures of two or more proximal long bones	
Instable thorax	
Pelvis fracture	
Amputation proximal of hand/foot	
Paraplegia after trauma	
Open skull fracture	
Burns of > 20% and grade >2a	

*: American College of Surgeons Committee on Trauma (ACS-COT) TTA criteria. GCS: Glasgow Coma Scale

### Study group

Between August and December 2020, 50 patients admitted to the TRR due to trauma mechanism criteria and without any injury-related TTA criteria were included in the study (Table 1) [7]. All patients had to meet the inclusion criteria and none of the exclusion criteria listed in Table 2. As patients with injury-related TTA criteria and severely injured trauma patients need immediately resuscitation, we excluded these patients from the study. All patients were included with consent of the trauma team leader, and informed written consent of the patient was provided right after becoming again capable of consenting. The study was approved by the Ethics Committee board, Faculty of Medicine, Heidelberg University, Heidelberg, Germany (No. S-223/2015). The study was conducted in accordance with the Helsinki Declaration.

**Table 2:** List of inclusion and exclusion criteria. TRR: trauma resuscitation room; ISS: injury severity score

Inclusion criteria	Exclusion criteria
Age ≥ 18	Age < 18
Primary admission to trauma resuscitation room of the study centre	Admission to another hospital and transfer into study centre
Admission solely based on trauma mechanism criteria (Table 1)	Admission to trauma resuscitation room based on critical vital signs (Table 1).
ISS < 16 at the end of TRR-treatment	ISS ≥ 16 at the end of TRR-treatment
Informed written consent of contractually capable patient within 24 h after admission	Not contractually capable patient within 24 h after admission

**Fig. 1:**
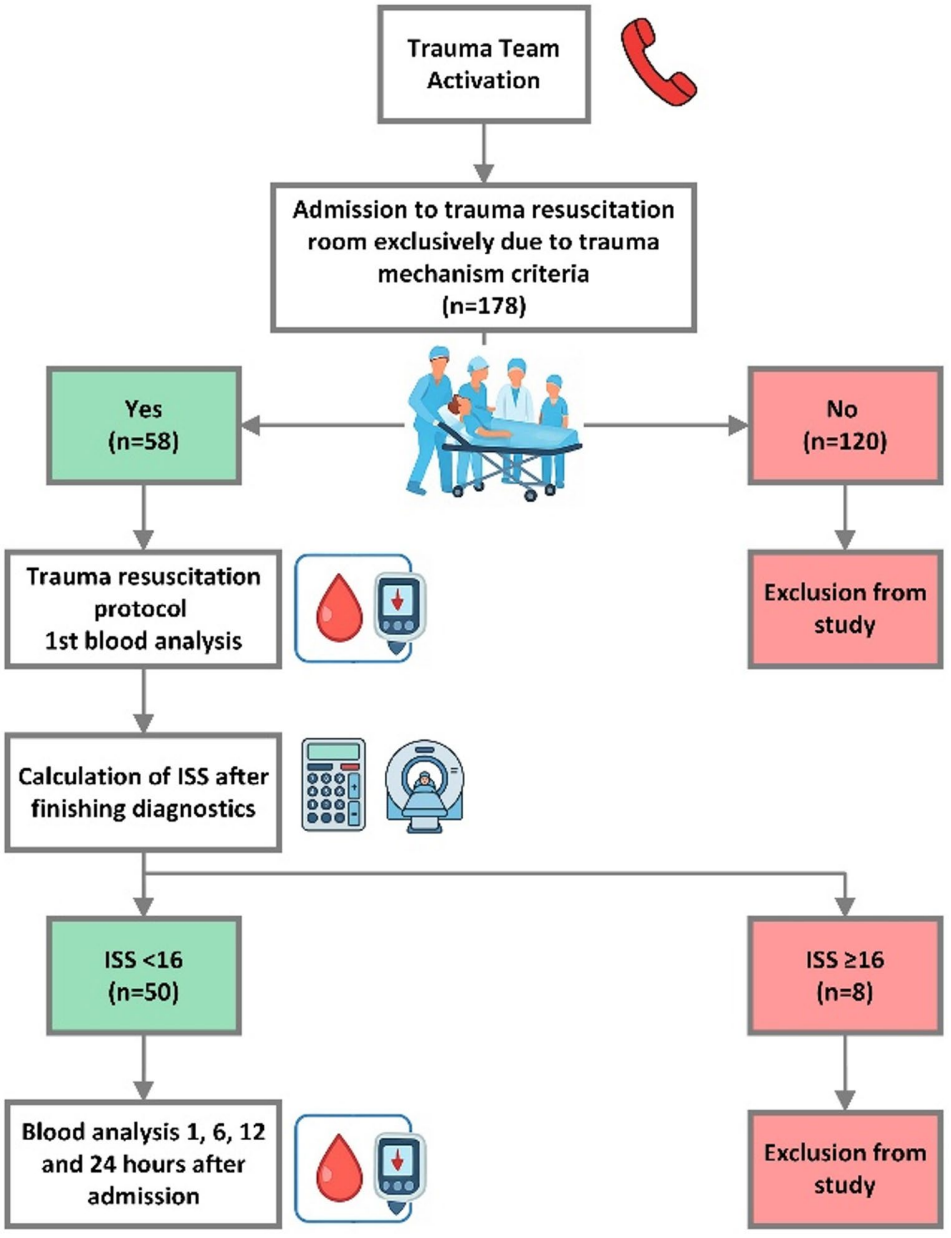
Flow chart about inclusion criteria used for study and study design

Blood samples of patients who met the inclusion criteria were collected immediately upon admission to TRR as well as 1, 6, 12 and 24 h after admission. IL-6 levels were measured using a fully automated immunoassay analyser with a standardized chemiluminescence assay and results were available after approximately 15 min. All blood tests, which were performed in this study, are listed in Table 8 in the appendix. Further medical and demographic data were collected from the hospital reporting system. Detailed information about the study design is displayed in Fig. 1.

As this study focused on differentiation between mildly and moderately injured patients, subjects with an ISS of ≥ 16 were excluded from this study. For further analysis, the study group was divided into two subgroups according to ISS (Mild: ISS 0–8, Moderate: ISS 9–15) [16]. Twenty-five healthy probands with informed written consent, who had not experienced any trauma within 4 weeks before blood sample collection, served as a control group for blood analysis to define a healthy baseline of measured blood values.

### Rule out protocols of moderately injured patients

To use IL-6 levels for a robust differentiation between mildly and moderately injured patients we performed a ROC-analysis of IL-6 concentration at time of admission to predict moderate injuries in the study population. In a first step, we defined IL-6 cut-off values at time of admission to rule out moderate injuries and patients with an IL-6 concentration below the cut-off value were categorized as mildly injured. Based on the results, we calculated over- and undertriage rates for this cut-off value. The prevalence of moderately injured trauma patients between January 2018 and December 2021 was used to calculate NPVs.

To evaluate whether a two-step rule out protocol based on determination of IL-6 concentrations at time of admission and 1 h after admission can further improve over- and undertriage rates, all patients with IL-6 concentrations above the cut-off value at time of admission were categorised as potentially moderately injured and therefore underwent analysis of IL-6 concentration 1 h after admission. Subsequently, we defined a cut-off value for IL-6 concentration 1 h after admission and patients with an IL-6 concentration below this cut-off value were also categorized as ruled out for moderate anatomical injuries.

### Statistics

For continuous data and scores, the empirical distribution was assessed by determining the mean, median, standard deviation, minimum, and maximum. Categorical data were described by their absolute and relative frequencies. Statistical data analysis of IL-6 levels of the distinct groups at the same timepoint was performed using the Mann-Whitney-U-test. Correlation analysis was executed with the Spearman correlation analysis. Within the ROC-analysis, we used the Youden index for defining optimal IL-6 levels. Cut-off values in the ROC-analysis were determined by the IL-6 concentration with a sensitivity of ≥ 0.9 and the highest specificity.

SPSS Statistics version 29 (IBM, Armonk, NY, USA) and R version 4.3.2 (The R Project for Statistical Computing, Vienna, Austria) were used for statistical analysis and visualisation. Graphics were created with Microsoft Office Version 2506 (Redmont, WA, USA) and Microsoft Copilot. The significance level was set at α = 0.05.

## Results

### Trauma mechanism-based TTA and prevalence of moderately injured patients in the TRR

**Fig. 2:**
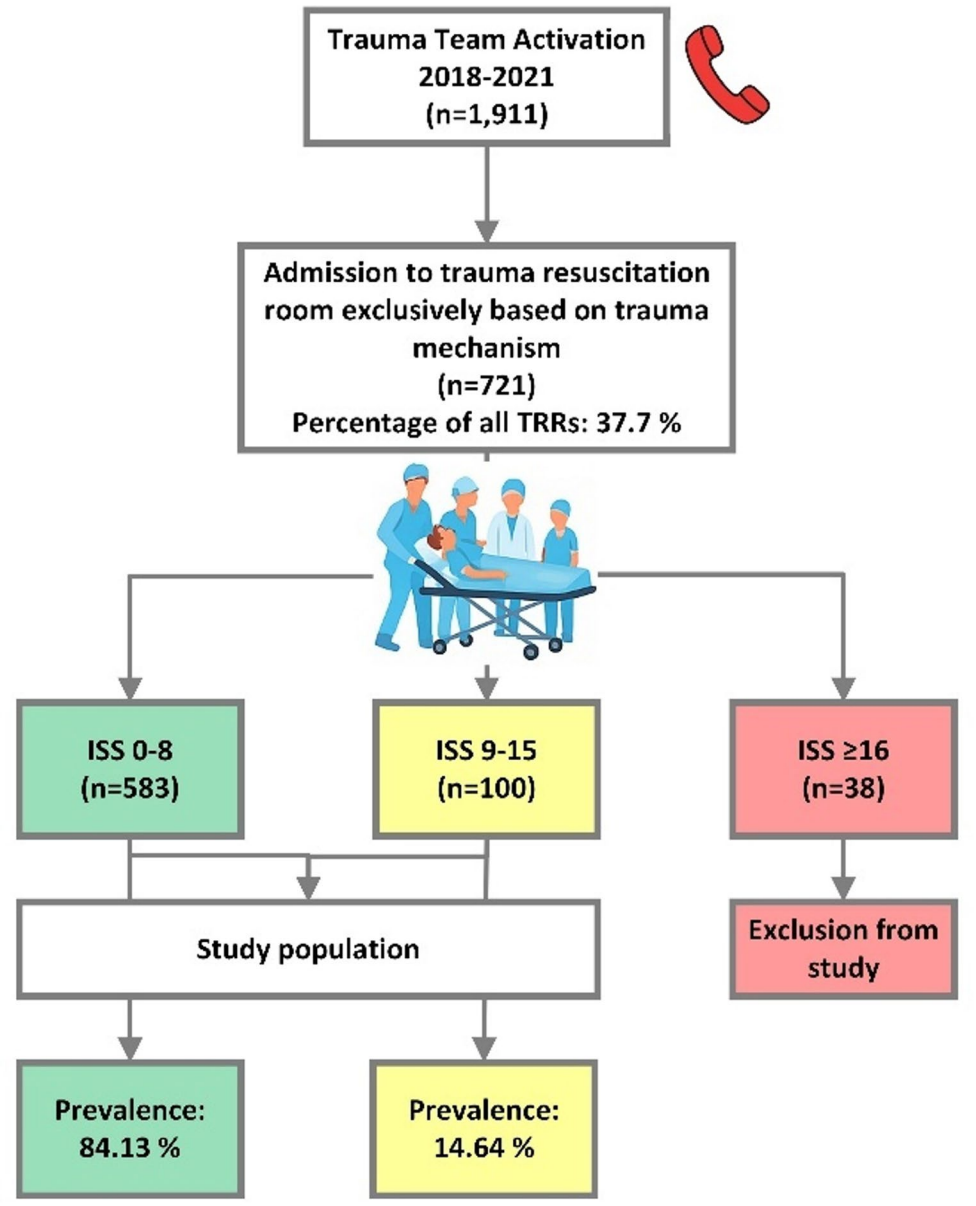
Flow chart about trauma mechanism-based trauma team activation and prevalence of mildly, moderately, and severely injured patients in the trauma resuscitation room (TRR)

Among 1,911 patients, who were admitted to the TRR of our hospital between 2018 and 2021, 721 patients (37.7%) triggered TTA solely based on trauma mechanism criteria without any injury-related criterion. As displayed in Fig. 2, 80.86% of these patients were mildly injured (ISS 0–8) and only 5.3% of these patients suffered from severe injuries, defined as ISS ≥ 16. Prevalence calculation of moderately injured trauma patients within the study population revealed a prevalence of 14.64%, while prevalence of mildly injured patients was 84.13%.

### Study group characteristics

Of the fifty patients in the study group, 31 (62%) were males and 19 (38%) were females. The average age was 49 years (interquartile range (IQR) 31–66 years). Average time between accident and admission to TRR was 56.7 min (IQR 42.0–73.5 min). No correlation between injury severity and time from trauma to admission was detected. Within the study group, an average ISS of 5.9 (IQR 1.6-9.0) was determined. Nineteen patients were moderately injured, while thirty-one patients sustained mild injuries. In four patients, no direct trauma sequelae were seen (ISS 0). Five patients underwent surgical therapy within the first 24 h (2x surgical treatment of proximal femur fracture, 2x external fixation of instable fractures (distal radius; Lisfranc injury), 1x plate osteosynthesis of a proximal humerus facture, 1x bursectomy), six patients after 24 h, but within the same hospital stay. Elective surgery after discharge was performed in three patients. No patient needed an emergency surgery. 31 patients (62%) were admitted to ICU or intermediate care unit (IMC) with a mean length of stay at ICU/IMC of 1.9 days (± 2.3 days). The most frequent indication for ICU admission was respiratory insufficiency (*n* = 10), followed by monitoring due to hospital standard (*n* = 8) and monitoring due to traumatic brain injury (TBI) (*n* = 7; others: *n* = 6). Within the group of patients, which were monitored at ICU due to hospital standards, solely one patient had an ISS ≥ 9. IL-6 level in this patient at time of admission was 5.6 pg/ml. Detailed information about mildly and moderately injured patients is provided in Table 3.

**Table 3:** Gender, Age, ISS and MAIS in the study population and distinct groups

Group	*n*	Sex Mean	Age Mean (IQR)	IL-6 at admission Mean (IQR) [pg/ml]	IL-6 1 h after admission Mean (IQR) [pg/ml]	ISS Mean (IQR)	MAIS Mean (IQR)
All	50	M: 31 (62%) F: 19 (38%)	49 (31–66)	11.65 (2.63–12.70)	19.34 (4.63–26.20)	5.9 (1.6-9.0)	1.72 (1.00-2.8)
Mild injuries (ISS 0–8)	31 (62%)	M: 14 (45%) F: 17 (55%)	45.6 (26.-62)	4.97 (< 2.0–6.9)	12.86 (3.43–22.6)	2.4 (1.0–4.0)	1.2 (1.0–2.0)
Moderate injuries (ISS 9–15)	19 (38%)	M: 17 (89%) F: 2 (11%)	54.7 (45–73)	22.78 (5.1–34.0)	30.75 (11.78–40.68)	11.5 (9.0–13.0)	2.5 (2.0–3.0)

IL-6: Interleukin-6; ISS: injury severity score; MAIS: maximum abbreviated injury scale; M: male; F: female; IQR: interquartile range

### Interleukin-6

**Fig. 3:**
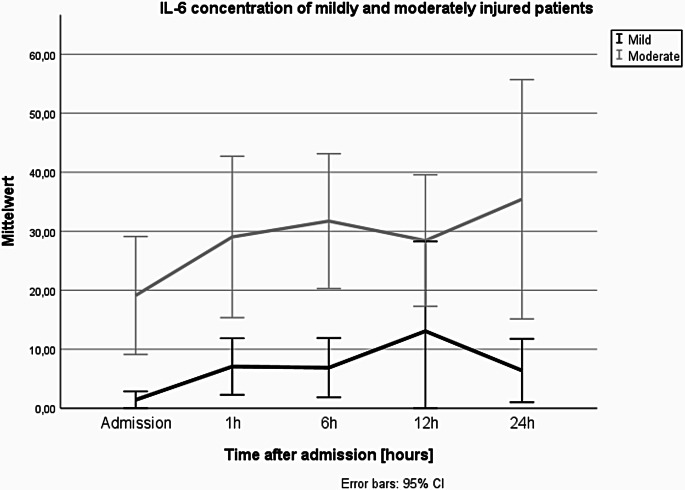
Mean IL-6 levels detected in mildly and moderately injured patients during the first 24 h after admission. Number of blood samples: *n* = 15–29 per group and timepoint

The IL-6 concentration in both groups showed an increase from admission to 6 h after initial blood sampling. Mean IL-6 concentration in the moderately injured group was significantly higher at all timepoints except 12 h after admission compared to IL-6 levels of mildly injured patients, as displayed in Fig. 3 (0 h: *p* = 0.002; 1 h: *p* = 0.004; 6 h: *p* = 0.019; 12 h: *p* = 0.076; 24 h: *p* = 0.049). In the moderately injured group, patients showed highest IL-6 levels after 6 h with a mean concentration of 31.34 pg/ml, while the highest IL-6 concentration in mildly injured patients was detected after 12 h with a mean concentration of 23.74 pg/ml.

For further analysis, we performed a Spearman correlation of ISS and IL-6 levels, which showed a significant positive correlation across all five timepoints. While time of admission, 1 h and 6 h after admission showed a strong correlation, IL-6 at later timepoints correlated moderately with ISS. The highest correlation was proven for blood collected right at admission (*r* = 0.664, *p* < 0.001), followed by timepoint 1 h after admission (*r* = 0.563, *p* < 0.001) (Table 4). As IL-6 rises quickly after trauma, we performed a correlation analysis of IL-6 at admission and the time elapsed from trauma to TRR admission and detected a significant positive correlation (*r* = 0.467, *p* = 0.004).

**Table 4:** Spearman correlation of ISS and IL-6 measured at different timepoints at and after admission

	At admission	1 h after admission	6 h after admission	12 h after admission	24 h after admission
*r*	**0.664**	**0.563**	**0.511**	**0.461**	**0.498**
p	< 0.001	< 0.001	< 0.001	0.003	0.002
n*	40	44	43	39	36

*: Although fifty patients were included in the study some samples could not be assessed due to technical or medical issues

The IL-6 concentration of healthy probands in the control group remained below the detection limit of 2.0 pg/ml in all participants. Among the twenty-five probands in the control group, ten were males and fifteen were females. The average age of the probands was 36.4 (± 15.3) years.

### Rule out protocols of moderately injured patients

**Fig. 4:**
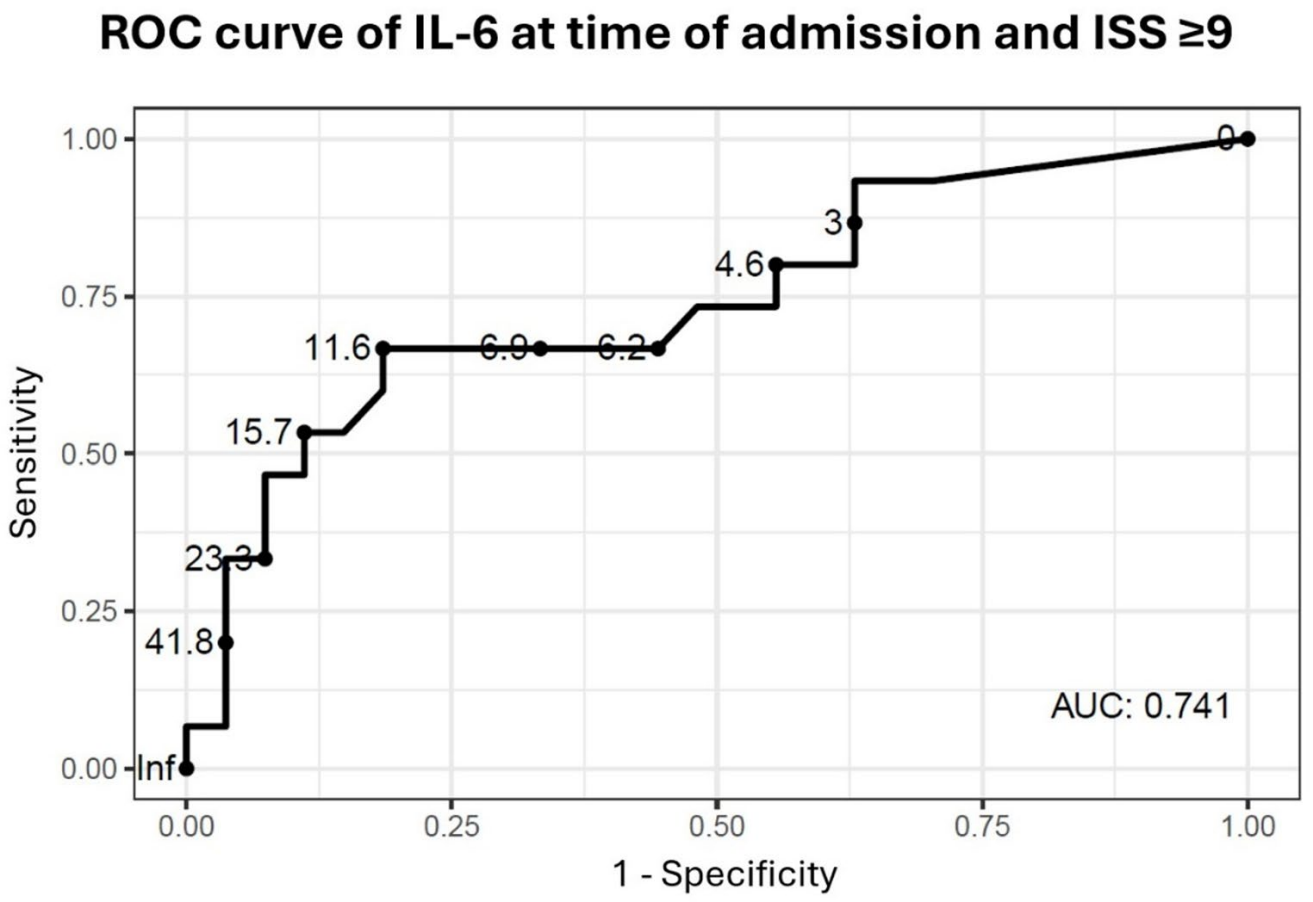
Receiver operating characteristics (ROC) curve of interleukin-6 (IL-6) at time of admission and injury severity with an injury severity score (ISS) ≥ 9. AUC: area under the curve

To evaluate whether IL-6 at time of admission can be used to differentiate between mild and moderate injuries we performed a ROC-analysis of IL-6 at time of admission to predict an ISS of ≥ 9 (Fig. 4). The ROC-analysis showed an AUC of 0.741. Highest Youden index was detected for IL-6 cut-off at 11.6 pg/ml with a sensitivity of 0.667 and a specificity of 0.815.

A sensitivity of ≥ 0.9 was required to preserve a sufficient patient’s safety during the rule out protocol. The IL-6 cut-off value of 2.9 pg/ml at time of admission showed the highest specificity with a sensitivity of ≥ 0.9 (sensitivity: 0.933; specificity: 0.370) to predict moderate injuries in the study group. The calculated NPV was 0.970. Therefore, we chose an IL-6 concentration of 2.9 pg/ml at time of admission as cut-off value to differentiate between mildly and moderately injured patients. Only one ruled out patient was moderately injured and therefore falsely identified as overtriaged, as displayed in Table 5. This patient presented at the TRR with a mild respiratory insufficiency with need of oxygen supply.

Evaluation of a two-step rule out protocol with IL-6 determination at time of admission and 1 h after admission was performed by ROC-analysis of the cohort, in which IL-6 levels ≥ 2.9 pg/ml were detected. This analysis showed highest NPV for an IL-6 cut-off concentration of 11.7 pg/ml. NPV was 0.961, sensitivity was 0.917 with a specificity of 0.353. Calculated overall sensitivity of the two-step rule out protocol was 0.786 with a specificity of 0.633.

**Fig. 5:**
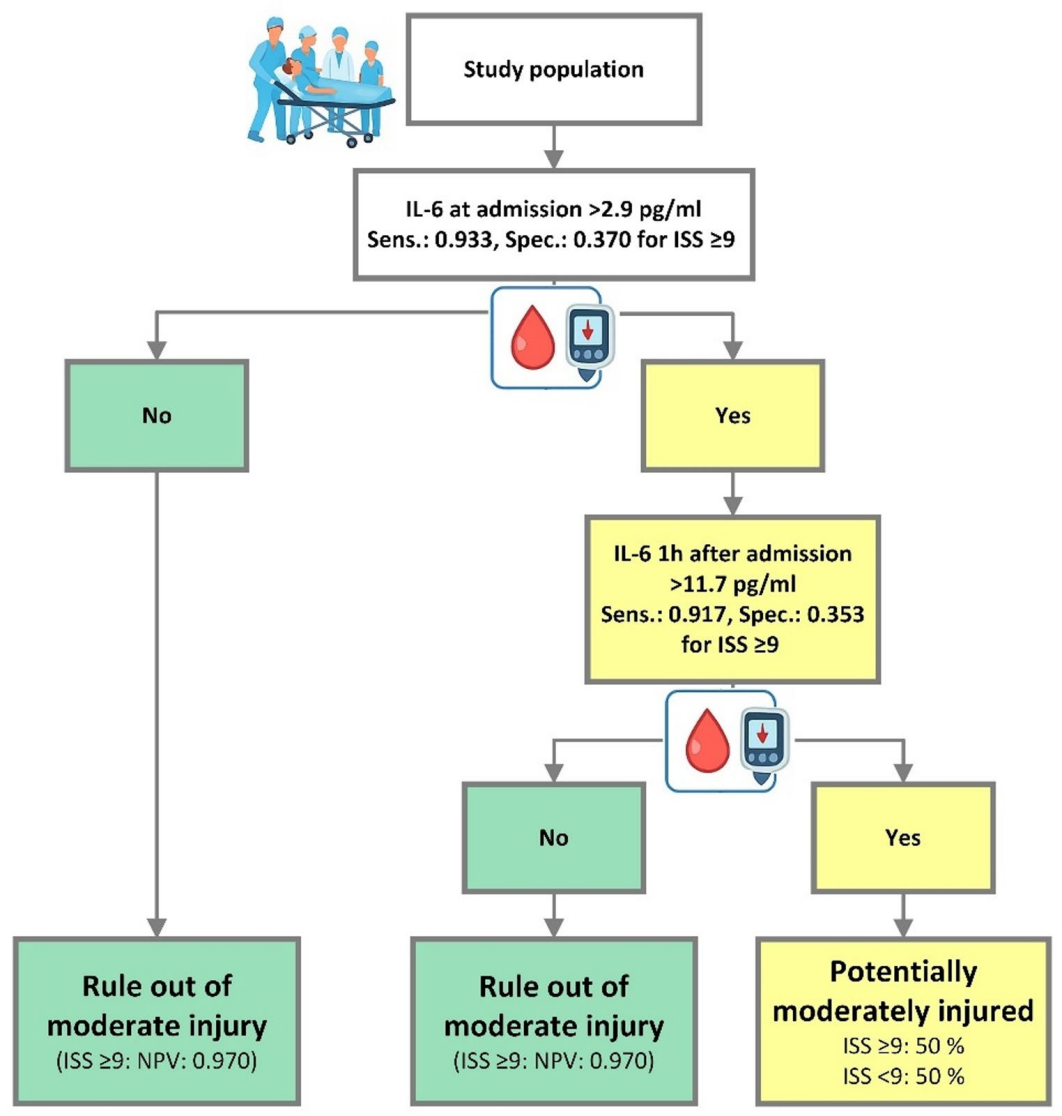
Flow chart of the two-step interleukin-6 (IL-6) combination test to rule out moderate injury severity in the study population. Sens.: Sensitivity; Spec.: Specificity; ISS: Injury Severity Score; NPV: negative predictive value

The fourfold table of IL-6 at admission and the two-step IL-6 combination test (Table 5), as well as the flow chart of the rule out protocol (Fig. 5) show that a rule out based on IL-6 concentration at time of admission identified only 2.4% of all patients as false “ruled out”. Therefore, calculated undertriage rate (rate of moderately injured patients, who were not identified as moderately injured) was 6.6% and overtriage rate was 54.8% (rate of mildly injured patients, who were not identified as mildly injured). The two-step rule out protocol identified 6.8% of all patients as false “ruled out” after combination of both tests and characterized 50% of all patients as mildly injured. While overtriage rate was lower than in the one-step protocol (50.0% vs. 54.8%), undertriage rate was clearly higher (21.4% vs. 6.6%).

**Table 5:** Fourfold table of interleukin-6 (IL-6) at admission and IL-6 combination test to predict ISS ≥ 9. Cut-off value for IL-6 at admission: 2.9 pg/ml

	IL-6 at admission		IL-6 combination test	
	IL-6 < 2.9 pg/ml	IL-6 ≥ 2.9 pg/ml	IL-6 negative*	IL-6 positive**
ISS < 9	10 (23.8%)	17 (40.5%)	19 (43.2%)	11 (25.0%)
ISS ≥ 9	1 (2.4%)	14 (33.3%)	3 (6.8%)	11 (25.0%)

*: IL-6 at admission < 2.9 pg/ml OR after 1 h < 11.7 pg/ml. **: IL-6 at admission ≥ 2.9 pg/ml AND after 1 h ≥ 11.7 pg/ml

### Potential reduction / early aborts of TRR treatments

**Fig. 6:**
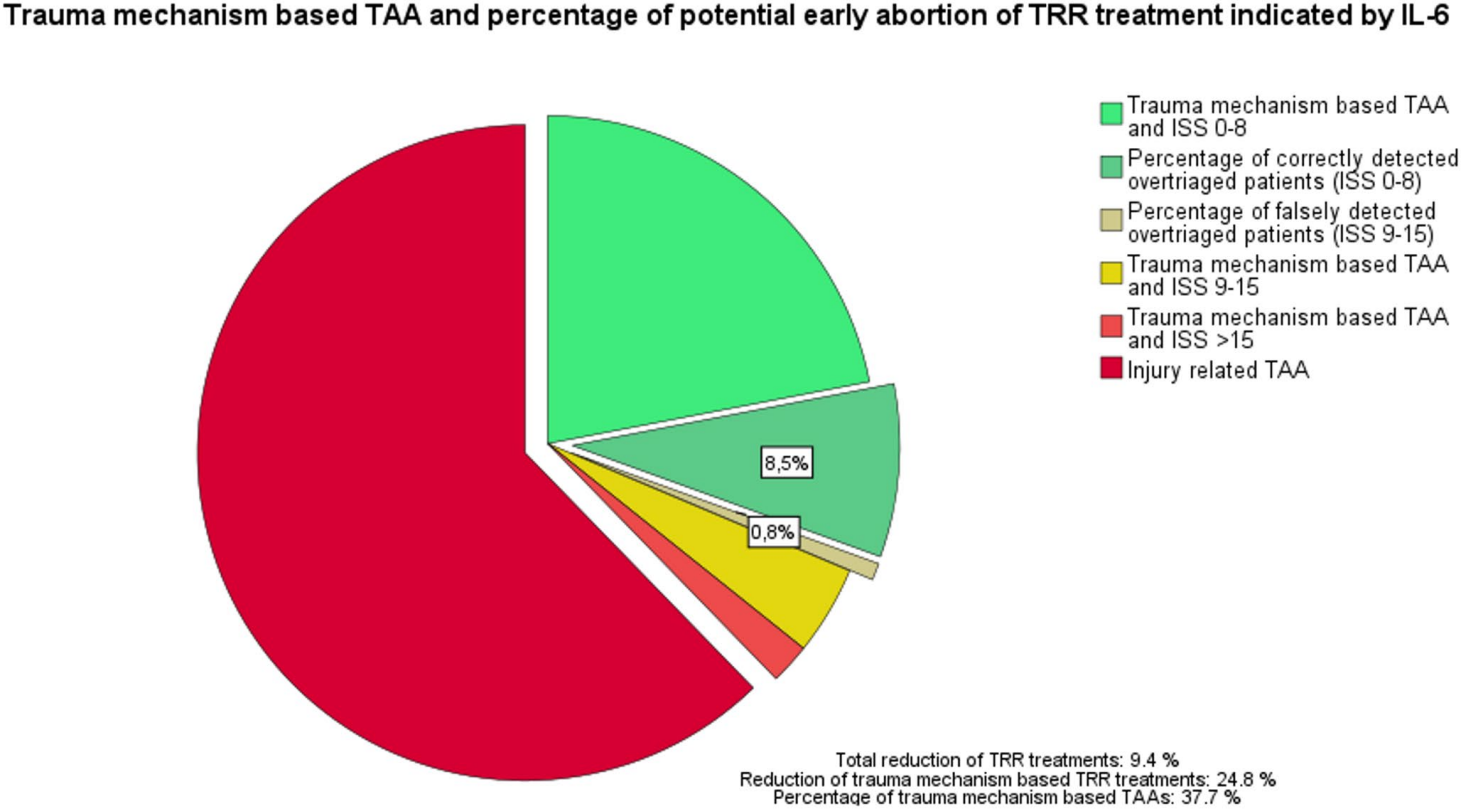
Pie chart of the percentage of trauma team activations (TTA) in the years 2018–2021. The separated pieces represent all TTAs based on trauma mechanism criteria. Highlighted pieces (dark green, dark yellow) indicate percentage of TRR treatments of potentially mildly injured patients with IL-6 levels < 2.9 pg/ml. ISS: injury severity score

Based on the prevalences of different criteria for TTA and the detected ISS of patients, who were admitted to the TRR due to trauma mechanism, we calculated the percentage of TRR treatments of anatomically mildly injured patients and therefore potentially overtriaged patients (displayed in light and dark green in Fig. 6). The combination of prevalences and ROC analysis of IL-6 levels at time of admission enabled us to calculate the percentage of TRR treatments, which would be identified as mildly injured based on IL-6 levels lower than 2.9 pg/ml. The implementation of an IL-6 cut-off value of 2.9 pg/ml at time of admission would reduce numbers of full TRR treatments and increase the number of early TRR treatment aborts of patients with solely trauma mechanism criteria by 24.8% and overall TRR treatments by 9.4%.

## Discussion

The early identification of mildly injured trauma patients and therefore overtriaged patients, if admitted to the TRR, is important to make TRR treatment more efficient and economical for a trauma centre. Although studies showed a correlation of IL-6 serum levels and injury severity in polytraumatised patients [21, 26–32], explicit data about correlation of IL-6 levels and injury severity in only mildly to moderately injured patients are missing. In addition, it was reported in multiple studies that IL-6 has a high predictive value for development of complications like sepsis, MODS or MOF and mortality, but we found no study, which evaluated the use of IL-6 to distinguish between mildly (ISS 0–8) and moderately injured trauma patients (ISS 9–15) as other studies focussed on patients with ISS of ≥ 9 or ≥ 16 [21–25].

Thus, to our knowledge this study is the first study, which evaluated whether IL-6 serum levels correlate with ISS in mildly (ISS 0–8) and moderately (ISS 9–15) injured patients, and whether IL-6 analysis is suitable for ruling out moderate anatomical injuries (ISS > 8) to support clinical decision making about overtriage and TRR treatment abortion. Especially in patients, who were admitted to the TRR solely based on trauma mechanism criteria, early IL-6 evaluation can be a promising additive tool to improve early differentiation of mildly and moderately injured trauma patients, as percentage of mild injuries in this cohort is high. An early and precise differentiation between mild and moderate injuries is of high clinical relevance as mortality rates in moderately injured patients are more than two times higher than in mildly injured patients and moderate injured patients can suffer from more severe injuries [16]. Point-of-care-testing (POCT) of IL-6 can measure results within 15 min and can therefore be easily integrated into the initial diagnostic assessment to improve the quality and efficiency of TRR treatment [33, 34].

Analysis of TTAs in our study between 2018 and 2021 showed that 37.7% of all TTAs based solely on trauma mechanism criteria. 80.9% of these patients were mildly injured, and another 13.9% were moderately injured resulting in only 5.3% severely injured patients. As ACS-COT seek for undertriage rates lower than 5% and overtriage rates lower than 50%, it is necessary to implement new strategies for early identification of overtriaged patients to have the option of an early abort of the TRR treatment to free resources, like POCT of biomarkers [8, 9].

This study reports that ISS correlates not only in severe and critical trauma or trauma in general as reported in other studies [21, 26–32], but also in anatomically mild and moderate trauma, with best correlation during early assessment at time of admission. In comparison to other biomarkers for early assessment of injury severity, which were analysed in other publications, IL-6 in our study showed a stronger correlation than copeptin, interleukin-10 (IL-10), creatinine, alkaline phosphatase, aspartate transaminase (AST) and alanine transaminase (ALT) [28, 35, 36]. Furthermore, IL-6 correlation with ISS in our study was comparable to IL-6 correlation in severe trauma patients what reflects the high potential for assessment of injury severity also in non-severe trauma patients [26].

Build on the strong correlation, ROC-analysis of IL-6 concentration at time of admission to predict an ISS of ≥ 9 in our study identified an IL-6 concentration of 11.6 pg/ml as the statistical best cut-off value (sensitivity: 0.667; specificity: 0.815). AUC and Youden index of this IL-6 concentration were comparable to studies, which utilized IL-6 to predict MODS after traumatic haemorrhagic shock [21] and sepsis in critical trauma patients (ISS ≥ 25) [25]. Although in our study IL-6 was not used to predict complications and mortality in severe and critical trauma patients, our results showed comparable predictive values of IL-6 in mildly to moderately injured patients. Therefore, we showed in this feasibility study that IL-6 is also a suitable tool for early differentiation between mild and moderate injuries and that IL-6 can serve, after adequate prospective pivotal validation, as a supportive diagnostic method in the multifactorial decision making of TRR treatment abortion. However, despite these promising results the clinical applicability should be validated on a representative cohort in a prospective study before it can be implemented with confidence in clinical routine.

Given that the objective of IL-6 concentration assessment in this study was to safely rule out moderate trauma severity (ISS > 8), a sensitivity of ≥ 0.9 for IL-6 levels at admission is justified. An IL-6 concentration of 2.9 pg/ml was the cut-off value with highest predictive power and a sensitivity ≥ 0.9 (sensitivity: 0.933; specificity: 0.370) in our study. As we found no study which evaluated the predictive power of a biomarker to predict moderate injuries, we compared our findings with other studies about biomarkers to predict severe injuries in trauma patients. In comparison to a study by Salvo et al. evaluating the serum biomarkers lactate and copeptin to predict severe injury with a high sensitivity of ≥ 95% and a specificity of 0.14 for lactate and 0.20 for copeptin, the predictive power of IL-6 in our study was higher, due to a higher specificity and an only slightly lower sensitivity [35]. A similar study by Hagebusch et al., who evaluated lactate to predict severe injury (ISS ≥ 16) in patients admitted to the TRR solely based on trauma mechanism criteria, reported a comparable AUC for lactate as we detected for IL-6 to predict moderate injuries [37].

Based on the underlying prevalence of 14.6% for moderately injured patients in our study population and a NPV of 0.970 for IL-6 of ≥ 2.9 pg/ml only one patient (2.4%) was falsely rule out and so falsely identified as mildly injured. This reflects the high predictive value and high level of patients’ safety of IL-6 determination at time of admission for moderate injuries. By ruling out moderate anatomical injuries in TRR patients with an IL-6 level below 2.9 pg/ml at time of admission IL-6 determination can support clinical decision about early abortion of TRR treatment of clinically unsuspicious patients at an early timepoint prior to extended diagnostics like whole body CT scan. Anatomically mildly injured patients suffer at the maximum from MAIS 2 in one anatomical location with MAIS 1 in two other anatomical locations, or MAIS 2 in two anatomical locations without any further injury in another anatomical location. Depending on the individual status, clinical appearance and comorbidities of the mildly injured patients, it can be considered to abort the TRR treatment and follow up on these patients in the regular emergency department with a reduced medical team. Therefore, identifying mildly injured patients by IL-6 determination at admission can reduce numbers of complete TRR treatments of patients with solely trauma mechanism criteria by 24.8% and overall complete TRR treatments by 9.4% as the TRR treatment could be potentially aborted at an early stage. Although overall number of TTA cannot be reduced, the potential to diminish completed TRR treatment including extended diagnostics with following ICU admissions for monitoring due to common institutional practices, can result in a significant reduction of human resources and financial burden, as TRR/ICU treatment costs are high and IL-6 POCT is a rapid and cheap diagnostic tool [33, 34, 38].

Although an IL-6 cut-off at 2.9 pg/ml at time of admission ruled out moderate injuries in 23.8% of all patients correctly, 54.8% of patients with IL-6 ≥ 2.9 pg/ml were only mildly injured. To further identify overtriage patients, which could be subject to early TRR treatment abort, we evaluated whether a two-step testing procedure with IL-6 determination 1 h after admission can improve predictive values for moderate injuries and can increase numbers of negative tested patients. The test combination rose the percentage of negative tested patients from 26.2% after the first test to 50% after the second test. This higher rate of negative tested patients resulted in a better false-positive rate of 25% and therefore reduced overtriage rates to 50%, but increased undertriage rates from 6.6% to 21.4% and so clearly above the recommended ACS-COT undertriage rate recommendation. Therefore, we do not recommend the two-step rule out protocol without other clinical assessment tools.

In general, a pre-test selection of the right patient is important, as the NPV depends on prevalence and any biomarker determination can delay resuscitation in seriously injured patients. Therefore, biomarkers to predict injury severity should be used exclusively in patients admitted to TRR based on trauma mechanism criteria, as risk of severe injury is lower in this group than in patients with TTA due to injury criteria. Like all biomarkers, IL-6 determination should always be used as an additive tool to support clinical decision making and not as a stand-alone criterion, as downgrading is a multifactorial process, which is based on several different parameters and assessments. It is important that patients eligible for IL-6 determination to rule out moderate injuries should show normal vital signs and should be able to be treated in the emergency department, as the patient should be transferred there if IL-6 is < 2.9 pg/ml. An unclear patient status, unstable or fluctuate vital signs, relevant comorbidities, a geriatric patient status and other risk factors for posttraumatic complications should be carefully evaluated, and in any situation where clinical stability is uncertain, IL-6–based decision-making should be avoided. Hence, the use of IL-6 determination at time of admission to support decision making for safe TRR treatment abortion of clinical stable patients based on other parameters can be beneficial. This is underlined by the fact that the only patient, in which IL-6 determination falsely ruled-out a moderate anatomical injury severity, presented with a moderate respiratory insufficiency, which was under the level of ACS-COT TTA criteria. Consequently, the combination of clinical assessment and IL-6 determination identified all anatomically moderately injured patients correctly.

Even though IL-6 concentration at time of admission correlates with elapsed time between trauma and admission, we still detected a good sensitivity and NPV for IL-6 at time of admission to predict moderate injuries in our study group (time between accident and arrival at TRR: 56.7 min (IQR 42.0–73.5 min)). As mean time between accident and admission at the hospital in Germany ranges between 60 and 66 min, this indicates that IL-6 measurements at time of admission are robust against time differences in most cases [39].

According to data of large trauma registers around the world male patients sustain trauma and undergo TRR treatment more often than females [39–41]. The sex distribution in our study was also not equal (62% male in our study), but representative for overall sex distribution in German TRRs, as the German TraumaRegister DGU^®^ reported a male sex in 69.9% of all cases over the last 10 years [39].

Due to high numbers of mildly injured patients, among patients treated in the TRR based on trauma mechanism criteria only, distribution between mildly and moderately injured patients was not balanced. In addition, the presented feasibility study is limited by the comparatively small case series of fifty patients, as it was designed retrospectively as a pilot study to evaluate, whether defining cut-off values of IL-6 to differentiate between mildly and moderately injured patients is possible.

Nevertheless, as this pilot study proofed that IL-6 at time of admission can retrospectively differentiate between mild and moderate injuries, it is a solid fundament for a subsequent prospective pivotal study with a far larger sample size to generate robust confirmatory data before this test can be implemented as a supportive marker into the clinical routine of downgrading algorithms. Therefore, a large multi-centre, prospective study for further evaluation of IL-6 to early identify overtriaged and only mildly injured patients admitted to TRR based on trauma mechanism criteria should be performed before this method can be recommended to be part of the TRR standard-of-care.

## Conclusion

This feasibility study shows retrospectively the ability of the biomarker IL-6 to differentiate between anatomically mildly (ISS 0–8) and moderately (ISS 9–15) injured trauma patients, who were admitted to the TRR solely based on trauma mechanism criteria. We found a significant correlation between injury severity, scored by ISS, and IL-6 within the first 24 h after admission of mildly and moderately injured patients treated in the TRR. Performed ROC-analysis of IL-6 levels at time of admission showed a high sensitivity and specificity to predict moderate injuries in the study population.

Based on our findings the IL-6 cut-off value should be set at 2.9 pg/ml at time of admission to identify mildly injured patients (ISS > 8) treated in the TRR as this value provided the highest predictive power with a sensitivity of ≥ 0.9. This IL-6 cut-off value enabled us to predict moderate injuries in the study population with a sensitivity of 0.933, a specificity of 0.370 and a NPV of 0.970. Using this IL-6 cut-off value resulted in undertriage rates of 6.6% and overtriage rates of 54.8%, which are close to ACS-COT recommendations for over- and undertriage. According to our data, a two-step rule out protocol does not lead to further clinical improvement, as overtriage rates were lowered only marginally to 50% but undertriage rates increased from 6.6% to 21.4%. Based on this first feasibility trial data, IL-6 at time of admission could be considered as an adjunctive parameter within a broader clinical framework to early identify overtriaged and only mildly injured patients in the TRR. However, IL-6 determination at time of admission should not be used as a standalone decision-making tool, as identified patients are subject to early TRR treatment abortion and to be further treated in the regular emergency department. Nevertheless, a prospective pivotal clinical trial is necessary to confirm our results before an implementation into clinical routine is possible.

## Supplementary Information

Below is the link to the electronic supplementary material.

Supplementary Material 1

## Data Availability

The data that support the findings of this study are not openly available due to reasons of sensitivity and are available from the corresponding author upon reasonable request.
