# Supplementary material for: Interleukin-6 to identify mildly injured patients in the trauma resuscitation room – a clinical feasibility study
Source: Eur J Trauma Emerg Surg. 2026 Mar 24;52(1):112. doi: 10.1007/s00068-026-03160-1 (PMC13013202; doi:10.1007/s00068-026-03160-1)
Supplement: Supplementary file 1 — Supplementary Material 1 [file 68_2026_3160_MOESM1_ESM.docx]

Supplementary File

Appendix

**Table 8** List of analysed blood tests

| **Blood analysis** | **Reference range** |
| --- | --- |
| Interleukin-6 | <15 pg/ml |
| C-reactive protein | <5 mg/l |
| Haemoglobin-concentration | 13 – 17 g/dl (males)  12 – 15 g/dl (females) |
| Erythrocytes | 4.3 – 6.1 /pl |
| Haematocrit | 0.38 – 0.52 |
| Mean corpuscular volume | 83 – 97 fl |
| Mean corpuscular haemoglobin | 27 – 33 pg |
| Mean corpuscular haemoglobin-concentration | 30 – 36 g/dl |
| Red cell distribution width | 12.9 – 18.7 % |
| Platelet count | 150 – 440 /nl |
| Hypochromic erythrocytes | 0 – 2 % |
| Leucocytes | 4 – 10 /nl |
| Sodium | 135 – 146 mmol/l |
| Potassium | 3.4 – 4.6 mmol/l |
| Calcium | 2.11 – 2.59 mmol/l |
| Urea | <45 mg/dl |
| Creatinine | 0.6 – 1.2 mg/dl |
| Glomerular filtration rate according to CKD-EPI (Chronic Kidney Disease Epidemiology Collaboration) | >60 |
| Glucose | 65 – 110 |
| Creatine kinase | <190 U/l |
| Creatine kinase – MB (muscle-brain type) | <6 % of creatine kinase |
| Troponin T, highly sensitive | <14 pg/ml |
| Glutamate-oxalacetate-transaminase | <46 U/l |
| Glutamate-pyruvate-transaminase | <50 U/l |
| Gamma-glutamyl-transferase | <60 U/l |
| Alkaline phosphatase | 40 – 130 U/l (males)  55 – 105 U/l (females) |
| Total bilirubin | <1,0 mg/dl |
| Pancreatic amylase | 8 – 53 U/l |
| Total protein | 60 – 80 g/l |
| Albumin | 30 – 50 g/l |
| Lipase | 19 – 63 U/l |
| Alcohol | Per mille |
| Prothrombin time | 70 – 125 % |
| International Normalised Ratio (INR) | <1,2 |
| Partial Prothrombin-Zeit | <35 s |
| Fibrinogen | 1,8 – 3,5 g/l |
| Thyroid-stimulating hormone (TSH) | 0,4 – 4,0 mU/l |
| pH value | 7,37 – 7,45 |
| Partial pressure of carbon dioxide | 37 – 50 mmHg |
| Partial pressure of oxygen | 36 – 44 mmHg |
| Base Excess | -2 – +3 mmol/l |
| Chloride | 95 – 105 mmol/l |
| Methaemoglobin | <1,5 % |
| Lactate | <16 mg/dl |
| Standard bicarbonate | 21 – 26 mmol/l |
| Actual Bicarbonate | 21 – 26 mmol/l |
| Oxygen saturation | 70 – 80 % |
